# Exploring the partial immunological mechanisms of hepatitis B virus reactivation in patients with hepatitis B and tuberculosis

**DOI:** 10.3389/fcimb.2026.1771585

**Published:** 2026-04-10

**Authors:** Chunhua Xiao, Zhenxiong Deng, Peng Xian, Xiuming Huang, Zhen Zou, Liyuan Long, Liang Han, Lubo Yang, Fei Shi

**Affiliations:** 1 Department of Infectious Diseases, Shenzhen People’s Hospital (The Second Clinical Medical College, Jinan University; The First Affiliated Hospital, Southern University of Science and Technology), Shenzhen, Guangdong, China; 2 Department of Infectious Diseases, Huizhou Third People’s Hospital, Guangzhou Medical University, Huizhou, Guangdong, China; 3 Department of Infectious Diseases, The Affiliated Huizhou Hospital, Guangzhou Medical University, Huizhou, Guangdong, China; 4 Department of Medical Laboratory,Huizhou Third People’s Hospital, Guangzhou Medical University, Huizhou, Guangdong, China

**Keywords:** drug-induced liver injury, HBV reactivation, hepatitis B virus, IFN-γ, tuberculosis

## Abstract

**Objective:**

To explore the immune changes caused by anti-tuberculosis treatment, especially the fluctuations of IFN-γ, and their role in HBV reactivation and related liver injury.

**Method:**

This was a retrospective study. Firstly, 162 patients with hepatitis B and tuberculosis co-infection (initial HBV-TB group) and 162 patients with simple hepatitis B infection (initial HBV group) who were hospitalized at the Third People’s Hospital of Huizhou from June 2016 to September 2025 were consecutively included for preliminary comparison. Secondly, from the initial cohort, a baseline comparable subgroup was selected based on age, gender, and baseline levels of alanine aminotransferase (ALT) and aspartate aminotransferase (AST) for immunological analysis (a total of 14 patients in the TB-HBV group and 11 patients in the HBV group).Additionally, within the HBV-TB group, according to whether they received preventive antiviral treatment, they were divided into an untreated subgroup (n=16) and a treated subgroup (n=20) to analyze the impact of treatment on prognosis.

**Result:**

The comparison of baseline data between the HBV-TB group and the HBV group showed that the proportion of males was higher in the HBV-TB group, and the levels of WBC, NEU, Mon and PLT were higher than those in the HBV group. However, the levels of HBsAg, HBVDNA, ALT, AST, Lym and RBC were lower than those in the HBV group (all P<0.05). Among HBV-TB patients who did not receive anti-HBV treatment, 81.25% of the patients(13 case)had HBV reactivation, and 43.75% of them (7case)had severe liver injury. The incidence of Drug-induced liver injury(DILI) and the rate of HBV reactivation in this group were both higher than those in the anti-HBV treatment group (all P<0.05). In terms of immune indicators, the counts of lymphocytes, total T cells and CD4+ T cells in the HBV-TB group were all lower than those in the HBV group, while the levels of IFN-γ and IL-6 were higher(all P<0.05).

**Conclusion:**

It is recommended that HBsAg screening be conducted for all tuberculosis patients before anti-tuberculosis treatment, and preventive antiviral treatment be given to individuals with positive HBsAg to reduce the risk of HBV reactivation.

## 1 Background

According to the “2024 Global Tuberculosis Report” released by the World Health Organization (WHO), there were approximately 10.8 million new tuberculosis (TB) cases worldwide in 2023, with an incidence rate of 134 per 100,000 population. Among these, China reported 741,000 new cases, corresponding to an incidence rate of 52 per 100,000 population, making it one of the high-burden countries for TB globally (World Health Organization, 2023). Meanwhile, viral hepatitis remains a major global public health challenge. The “WHO 2022 Global Hepatitis Report” estimated that around 296 million people worldwide are living with chronic hepatitis B virus (HBV) infection (Polaris Observatory Collaborators, 2023). As a moderately endemic area for hepatitis B, China has included the hepatitis B vaccine in its national immunization program since 1992. However, the positivity rate of hepatitis B surface antigen (HBsAg) among the adult population remains relatively high, approximately 8.00% (Liu et al., 2016). The dual epidemic of TB and hepatitis B poses a severe threat to the health of Chinese residents and imposes a heavy socioeconomic burden.

Anti-tuberculosis treatment requires long-term multi-drug combination therapy, which often induces various adverse reactions. Among these, drug-induced liver injury(DILI)is the most common and severe (Lönnroth et al., 2010). This risk is particularly prominent in patients with concurrent HBV infection. Previous studies have shown that the incidence of DILI in this population is 2–5 times higher than in non-HBV-infected individuals (Zheng et al., 2014; National Clinical Research Center for Infectious Diseases Jiangxi Branch Jiangxi Provincial Key Laboratory of Tuberculosis, 2025). Notably, in stark contrast to this well-established high risk, systematic HBV screening and intervention before initiating anti-tuberculosis treatment have not yet become standard clinical practice (Lönnroth et al., 2010; Liaw et al., 2012), leaving this group of patients exposed to a high risk of liver failure and even death (Chen et al., 2018). The mechanisms underlying the increased susceptibility of such patients are complex. In addition to the inherent direct hepatotoxicity of drugs such as isoniazid (e.g., interfering with metabolism and inducing oxidative stress) (Ramappa and Aithal, 2013; Yew et al., 2018), the WHO guidelines also explicitly state that drugs like rifampicin can cause HBV reactivation through immune suppression (World Health Organization, 2025).

Both tuberculosis (TB) and hepatitis B virus (HBV) infection can induce immune abnormalities. When coexisting in the same host, their immune interactions may significantly affect clinical outcomes. Whether anti-tuberculosis treatment disrupts this fragile immune balance, thereby inducing HBV reactivation and accelerating liver injury, has become a new clinical research hotspot in recent years. A study by Gao et al (Gao and Song, 2019). showed that anti-tuberculosis drugs promote active HBV replication, leading to a state of inflammation in the body, which alters liver metabolic function and increases the hepatotoxicity of drugs. Puga et al (Puga et al., 2019). reported that in incarcerated populations with concurrent HBV infection, combined antiviral therapy during anti-tuberculosis treatment can effectively reduce the risk of DILI. However, systematic studies on the dynamic changes in immune status and viral replication levels during the treatment of HBV-TB co-infected patients are currently scarce. To explore the potential mechanisms underlying their synergistic pathogenicity, this retrospective study systematically compared multiple clinical indicators between HBV-TB co-infected patients and those with isolated HBV infection. We aim to clarify the baseline viral load characteristics of HBV-TB patients, depict the dynamic change trajectories of immune indices and HBV-DNA levels during anti-tuberculosis treatment, and further explore the partial immunological mechanisms underlying HBV reactivation, thereby providing empirical evidence for optimizing clinical intervention strategies and effectively reducing the risk of liver damage.

## 2 Methods and patients

### 2.1 Research design and patients

This study is a retrospective study. Firstly, 162 patients with hepatitis B and tuberculosis co-infection (initial HBV-TB group) and 162 patients with simple hepatitis B infection (initial HBV group) who were hospitalized at the Third People’s Hospital of Huizhou from June 2016 to September 2025 were consecutively included for preliminary comparison. Secondly, to more reliably compare the immunological differences between the two groups, we selected a baseline comparable subgroup from the initial cohort based on age, gender, and baseline ALT and AST levels. Finally, a total of 14 patients in the HBV-TB group and 11 patients in the HBV group were included in the immunological analysis. Additionally, within the HBV-TB group, according to whether they received preventive antiviral treatment, they were divided into an untreated subgroup (n=16) and a treated subgroup (n=20) to analyze the impact of treatment on prognosis.

Inclusion criteria: ① Age ≥18 years old; ② In line with “Guideline for primary care of pulmonary tuberculosis (2018)” (Chinese Medical AssociationChinese Medical Journals Publishing House et al., 2019) and “WHO consolidated guidelines Diagnostic criteria for active pulmonary tuberculosis in Module 3 of “Tuberculosis” (World Health Organization, 2025) and positive HBsAg (HBV-TB group); ③ Patients with positive HBsAg. Exclusion criteria: Patients co-infected with HAV, HCV, HDV, HEV, HIV, autoimmune hepatitis, alcoholic liver disease, liver cancer, those who have received anti-HBV treatment within half a year, those who have received anti-tuberculosis treatment, and those with incomplete data.

The study was approved by the Ethical Committee of the Huizhou Third People’s Hospital(Ethical Number:2025-KY-154-01), which waived the need for informed consent because all the data used in this retrospective study were routinely obtained and no additional procedures were carried out.The study adhered to the principles of the Declaration of Helsinki.

### 2.2 Data gathering

General demographic data, clinical data and laboratory results were the main data types collected in this study, including age, gender, prognosis, alanine aminotransferase (ALT), aspartate aminotransferase(AST),alkaline phosphatase(ALP),Total Bilirubin(TBIL),Direct Bilirubin(DBIL), Albumin(ALB),Globulin(GLB),HBVDNA,Hepatitis B Surface Antigen(HBsAg),Hepatitis B e Antigen(HBeAg), white blood cell (WBC),lymphocyte (Lym), monocyte(Mon),neutrophil(NEU), red blood cell(RBC),Hemoglobin(HGB), platelet(PLT);interferon-γ(IFN-γ), natural killer(NK), inerleukin (IL),tumor necrosis factor(TNF) and other lymphocyte subsets and cytokines.

### 2.3 Diagnosis of DILI and HBV reactivation

Diagnostic criteria for DILI: During anti-tuberculosis treatment, patients ①ALT≥3 times the upper limit of the normal value (ULN) and/or TBil≥2ULN; ②AST, ALP and TBil all increase simultaneously, and at least one of them is ≥2 times ULN, especially when accompanied by elevated GGT and excluding bone diseases and bile duct diseases (Chinese Medical Association Tuberculosis Branch, 2024); ③ If the patient does not meet the above two criteria, but the RUCAM (Roussel Uclaf Causality Assessment Method) system score is ≥3 points, DILI can also be diagnosed (Mao et al., 2024).

HBV reactivation (HBV-R) refers to the clinical manifestations of sudden increase in HBV replication, decompensation of liver function or acute liver failure in inactive or recovered HBV-infected individuals (Reddy et al., 2015; Bersoff-Matcha et al., 2017; European Association for the Study of the Liver, 2025).

### 2.4 Statistical analysis

The data were statistically analyzed using SPSS 25.0 and GraphPad Prism 8.0.1 software. The continuous variables with normal distribution were expressed as mean plus or minus standard deviation. The independent sample t-test was used for comparison between the two groups. The continuous variables with non-normal distribution were expressed as median (25th quard, 75th quard). The Mann−Whitney rank sum test was used for comparison between the two groups. Counting data were expressed as the number of cases (rate), and chi-square test was used for comparison between groups. A two-sided P-value < 0.05 was considered statistically significant. Furthermore, due to the small sample sizes of some subgroups in this study, we mainly focused on the effect size and descriptive trends, rather than overly relying on p-values.

## 3 Result

According to the inclusion criteria, a total of 162 patients with HBV-TB (HBV-TB group) were included in this study as the case group, and 162 patients with isolated hepatitis B virus infection during the same period were randomly matched at a ratio of 1:1 as the control group (HBV group).

### 3.1 Comparison of baseline clinical data and laboratory test results between the HBV-TB group and the isolated HBV group

The proportion of males in the HBV-TB group was higher than that in the HBV group, and there was no significant difference in the average age between the two groups. The levels of HBsAg, HBVDNA, ALT, AST, Lym and RBC in the HBV group were higher than those in the HBV-TB group. The levels of WBC, NEU, Mon and PLT in the HBV-TB group were higher than those in the HBV group, and the differences were statistically significant (all P<0.05). Shown in **Table 1**.

**Table 1:** Comparison table of baseline-related indicators between two groups.

Variable	Classification	HBV-TB group (n=162)	HBV group (n=162)	χ^2^/z/t	*p*
Sex,n (%)					
	male	129 (79.63)	93 (57.41)	18.544	<0.001
	female	33 (20.37)	69 (42.59)		
Age,years		50.23 ± 16.27	52.54 ± 16.13	-1.282	0.201
HbsAg,IU/mL		802.65 (216.20,1806.90)	1346.17 (115.15,3424.54)	-2.040	0.041
HBeAg-negative,n (%)		144 (88.89)	142 (87.65)	0.119	0.730
HBVDNA,IU/mL		1450.00 (135.90,30557.50)	4425.00 (647.00,192250.00)	-1.992	0.046
ALT,U/L		20.00 (12.75,35.00)	24.40 (19.00,38.00)	-3.651	<0.001
AST,U/L		23.00 (17.00,32.00)	24.00 (20.00,33.15)	-2.201	0.028
WBC,*10^9^/L		7.60 (6.03,9.03)	6.20 (5.28,7.73)	-4.282	<0.001
Lym,*10^9^/L		1.20 (0.90,1.60)	1.80 (1.36,2.30)	-7.646	<0.001
Mon,*10^9^/L		0.70 (0.50,1.00)	0.50 (0.40,0.65)	-5.653	<0.001
Neu,*10^9^/L		5.35 (3.90,6.83)	3.60 (2.90,4.63)	-6.849	<0.001
RBC,*10^12^/L		4.23 ± 0.67	4.50 ± 0.81	-3.259	0.001
PLT,*10^9^/L		294.50 (227.75,402.75)	216.50 (173.75,257.00)	-8.156	<0.001

### 3.2 Comparison of relevant indicators between the non-anti-HBV treatment group and the anti-HBV treatment group of HBV-TB patients

Among the 162 cases of HBV-TB included in this study, 16 cases did not receive anti-HBV treatment during anti-tuberculosis treatment. By comparing before and after anti-tuberculosis treatment, it was found that the HBV DNA of 12 cases (75.00%) showed an upward trend after anti-tuberculosis treatment. In addition, HBV reactivation occurred in 13 cases (81.25%) of the patients, among which 7 cases (53.85%) suffered from liver injury, liver failure or decompensation of liver function due to reactivation. It is shown in **Table 2**. We randomly selected 20 patients who received anti-HBV treatment and compared them with the non-anti-HBV group. The results showed that there was no statistically significant difference in age and gender between the two groups (p>0.05). The incidence of DILI, HBV reactivation rate, HBVDNA level and ALT level in the non-anti-HBV group were all higher than those in the anti-HBV group, while the platelet level in the anti-HBV group was higher than that in the non-anti-HBV group. The differences were statistically significant (all p<0.05), which are shown in **Table 3**.

**Table 2:** shows the HBVDNA levels and clinical outcomes of 16 patients who did not receive anti-HBV treatment before and after anti-tuberculosis treatment.

Case	Sex	Age (years)	Before anti-tuberculosis treatment	After anti-tuberculosis treatment	HBVDNA(IU/ml)		Prognosis
					Before	after	
case1	Female	37	2020/8/16	2021/2/18	<100	685.9	
case2	Male	32	2016/3/26	2016/7/16	4572	2135000	DILI
case3	Female	74	2018/4/12	2018/6/6	672	7533	DILI
case4	Male	28	2018/7/25	2018/9/27	47170000	187200000	DILI
case5	Female	72	2021/8/7	2021/10/9	412.7	1199	
case6	Male	63	2022/7/11	2022/9/7	1261000	85370000	DILI
case7	Male	71	2023/8/4	2024/4/7	5803	13100	
case8	Male	31	2018/7/5	2018/7/10	13950	23680	
case9	Male	30	2020/3/19	2021/5/6	<100	415.2	
case10	Male	59	2023/11/30	2024/7/10	<100	129000	
case11	Male	52	2023/7/17	2024/9/27	1244	1770	DILI, liver cirrhosis progresses from the compensated stage to the decompensated stage
case12	Male	49	2021/5/23	2022/3/2	2186	39230000	DILI, chronic hepatitis B progresses to the decompensated stage of liver cirrhosis
case13	Male	68	2017/1/18	2017/6/10	438800	251500	DILI, fulminant liver failure
case14	Male	39	2020/10/20	2020/11/16	288	232.4	
case15	Female	52	2022/5/9	2022/8/11	4210	976	
case16	Male	72	2021/2/24	2021/5/5	<100	<100	

**Table 3:** Comparison of relevant indicators between the anti-HBV treatment group and the non-anti-HBV group in HBV-TB patients.

Variable	Classification	No anti-HBV treatment (n=16)	Anti-HBV treatment (n=20)	*t/χ^2^/z*	*p*
Age,years		52.31±17.14	51.40±17.43	0.157	0.876
Sex,n (%)	Male	12 (75.00)	17 (85.00)	0.567	0.451
	Female	4 (25.00)	3 (15.00)		
DILI,n (%)	No	9 (56.25)	18 (90.00)	—	0.049^#^
	Yes	7 (43.75)	2 (10.00)		
HBV reactivation,n (%)	No	3 (18.75)	18 (90.00)	18.57	<0.001
	Yes	13 (81.25)	2 (10.00)		
HBVDNA IU/mL		10316.50 (758.43,1664125.00)	505.00 (100.00,11391.75)	-2.375	0.018
ALT,U/L		24.00 (15.00,66.75)	15.50 (15.00,17.00)	-2.045	0.041
AST,U/L		31.00 (22.25,61.50)	22.50 (21.00,28.25)	-1.802	0.071
TBIL,μmol/L		9.60 (6.75,20.10)	8.90 (5.30,10.20)	-0.940	0.347
DBIL,μmol/L		4.30 (2.90,8.25)	4.19 (2.15,7.02)	-0.796	0.426
ALB,g/L		32.05 (28.68,39.68)	33.75 (31.45,40.20)	-1.274	0.203
GLB,g/L		30.15 (25.83,34.08)	31.45 (26.75,34.30)	-0.016	0.987
WBC,*10^9^/L		6.35 (5.20,9.85)	6.80 (5.75,8.80)	-0.351	0.726
Lym,*10^9^/L		1.20 (0.85,1.60)	1.20 (1.10,1.55)	-0.609	0.542
Mon,*10^9^/L		0.65 (0.33,0.88)	0.60 (0.50,0.80)	-0.080	0.936
Neu,*10^9^/L		4.45 (3.35,6.10)	4.72 (3.08,6.48)	-0.064	0.949
RBC,*10^12^/L		4.29 (3.49,4.94)	3.85 (3.33,4.61)	-0.764	0.445
HGB, g/L		111.00 (104.25,136.50)	111.00 (89.25,124.25)	-0.956	0.339
PLT,*10^9^/L		228.00 (173.25,329.50)	292.00 (221.00,386.75)	-2.134	0.033

# Fisher’s exact probability method is adopted.

### 3.3 Comparison of lymphocyte subsets and cytokines between the HBV-TB group and the HBV group

**Figure 1:**
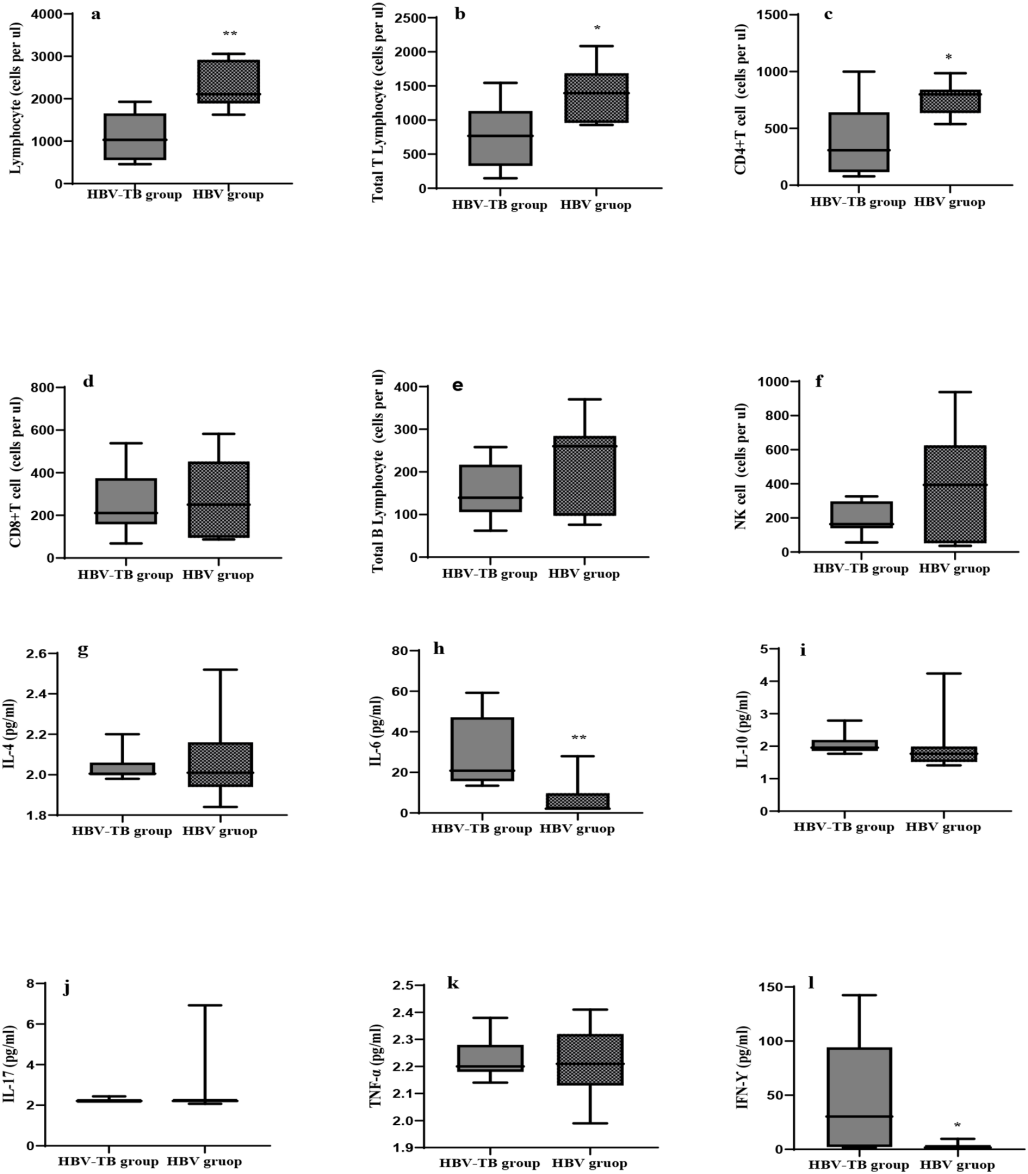
Comparison chart of two groups of lymphocyte subsets and cytokines. "*"indicate compared with HBV-TB group p<0.05, "**"indicate compared with HBV-TB group p<0.001.

To explore the immunological mechanism of a high proportion of elevated HBV DNA and reactivation in HBV-TB patients after receiving anti-tuberculosis treatment, we compared and analyzed the immunological characteristics of the HBV-TB group (n=14) and the HBV infection group (n=11). There was no statistically significant difference in gender,age,AST,and ALT between the two groups of patients, and they were comparable (**Table 4**). In the analysis of lymphocyte subsets (**Figures 1A–F**), the HBV-TB group exhibited a significant immunosuppressive state, specifically manifested as the counts of lymphocytes, total T lymphocytes,CD4+ T lymphocytes, the counts of CD8+ T lymphocytes, B lymphocytes, and NK cells in this group being lower than those in the HBV group (**Figures 1a-f**).

**Table 4:** Comparison of baseline data between the two groups of patients.

Variable	Classification	HBV-TB group (n=14)	HBV group (n=11)	*χ^2^/z*	*p*
Sex					
	Male	12 (85.71)	8 (72.73)	—	0.623
	Female	2 (14.29)	3 (27.27)		
Age,years		57.36±16.56	49±12.04	1.405	0.173
ALT, U/L		39.00 (28.00,60.75)	35.50 (25.00,50.25)	-1.344	0.179
AST, U/L		53.00 (38.00,65.00)	40.00 (32.00,55.00)	0.904	0.373

In the cytokine profiling analysis (**Figures 1g-k**), we observed that the levels of IFN-γ and IL-6 in the HBV-TB group were higher than those in the HBV group (**Figures 1l, h**). The levels of other cytokines, including IL-4, IL-10, TNF-α, and IL-17 (**Figures 1g, i, k, j**), showed little difference between the two groups.

## 4 Discussion

In the HBV-TB group, the prevalence was higher in males, consistent with the 2024 Global Tuberculosis Report (World Health Organization, 2023), likely related to higher rates of occupational exposure, smoking, alcohol consumption, and delayed healthcare-seeking behavior.Baseline data showed that over 80% of patients in both groups were HBeAg-negative. Compared to the isolated HBV group, the HBV-TB group had significantly lower HBsAg and HBV DNA levels, with >50% having HBV DNA below 2000 IU/mL. Although median ALT and AST were within normal ranges in both groups, they were significantly lower in the co-infection group. These characteristics—HBeAg negativity, low HBV DNA (<2000 IU/mL), and normal ALT—are consistent with “HBeAg-negative chronic infection” as defined by the natural history of chronic HBV infection (European Association for the Study of the Liver, 2025), suggesting that most HBV-TB patients may be in this phase before anti-tuberculosis treatment.However, for HBV-TB patients, antiviral therapy decisions should not rely solely on HBV natural history staging. Although immediate antiviral therapy is generally not recommended for HBeAg-negative chronic infection in isolated HBV (European Association for the Study of the Liver, 2025), active tuberculosis constitutes a special circumstance requiring re-evaluation. The “Expert Consensus on the Treatment of Pulmonary Tuberculosis Complicated with Chronic Hepatitis B Virus Infection” (National Clinical Research Center for Infectious Diseases Jiangxi Branch Jiangxi Provincial Key Laboratory of Tuberculosis, 2025) clearly recommends prophylactic antiviral therapy for HBV-TB patients, even if conventional treatment criteria are not met, to reduce drug-induced liver injury risk and ensure successful anti-tuberculosis treatment.

The results of this study show that patients who did not receive anti-HBV treatment had significantly increased HBV reactivation rate, viral load and the incidence of drug-induced liver injury. This discovery is similar to previous studies, revealing the key role of preventive antiviral treatment. Bivigou-Mboumba et al (Bivigou-Mboumba et al., 2018)reported that the virology response rate (undetected HBV DNA rate of 72.3%) of HBV-TB patients who received preventive antiviral treatment was significantly better than that of the untreated group (57.1%), confirming the effectiveness of the treatment from the virology level. Furthermore, several studies have provided corroboration from the perspective of liver injury risk: A meta-analysis conducted by Wei X et al (Wei et al., 2025). indicated that HCV co-infection could increase the risk of DILI in tuberculosis patients by 3.5 times; Trigo et al (Trigo et al., 2016). further confirmed that HBV infection itself is an independent predictor of DILI during anti-tuberculosis treatment. The above-mentioned studies collectively indicate that under the immune perturbation caused by anti-tuberculosis treatment, concurrent anti-HBV treatment may directly inhibit viral replication and block the “mixed liver injury” driven by HBV reactivation from the source.

IL-6 plays a core regulatory key in the inflammatory response triggered by tuberculosis. It is not only a marker of disease activity but also regarded as an independent predictor of poor treatment outcomes (Gupte et al., 2022). It has been reported in research that this inflammatory environment triggered by tuberculosis infection and its key factor IL-6 have also been found to have inhibitory effects on HBV replication under both *in vitro* and specific *in vivo* conditions (Palumbo et al., 2015). During this inflammatory process, innate immune cells are rapidly mobilized, and neutrophils act as first-line responders, directly participating in the bactericidal process by releasing reactive oxygen species and reactive nitrogen mediators (Santos et al., 2025); Meanwhile, monocytes, which act as precursors of macrophages, are also activated after the invasion of Mycobacterium tuberculosis and further participate in the immune response. These activated immune cells further secrete more inflammatory factors, positively amplifying the entire immune response in a feedback loop, which together constitute the typical inflammatory pathological process of tuberculosis. This complex inflammatory network has been clearly presented in clinical tests. The data from this study show that compared with the HBV group, the levels of IL-6, neutrophils and monocytes were elevated in the TB-HBV group, suggesting the activation of *in vivo* anti-tuberculosis immunity. However, while tuberculosis infection drives immune activation, it is also a wasting disease that is prone to cause anemia (Luies and du Preez, 2020), which explains the phenomenon of low red blood cell count in the HBV-TB group. It is worth noting that this active inflammatory state also stimulates platelet production. In this study, the platelet count in the HBV-TB group was higher, which was consistent with that reported by Kirwan et al (Kirwan et al., 2021).

Tuberculosis is not only a bacterial infectious disease, but also a complex immune-related disease. Patients with active pulmonary tuberculosis often present with cellular immune dysfunction, manifested as a decrease in the numbers of CD3^+^, CD4^+^ T cells and NK cells, an inversion of the CD4^+^/CD8^+^ ratio, while the proportion of CD8^+^ T cells is relatively elevated (Flynn et al., 1993). This study also found that compared with patients who were only infected with hepatitis B virus (HBV), patients who were infected with both hepatitis B virus and tuberculosis had lower total lymphocyte counts, total T lymphocyte counts, and CD4^+^ T lymphocyte counts. This might be related to the extensive inhibitory effect of tuberculosis infection on the overall immune status of the body. It is worth noting that the IFN-γ levels of patients in the HBV-TB group in this study were higher than those in the single HBV group, which is consistent with the results of Chen et al.’s study (Chen et al., 2009). Based on the known immunological principle-that tuberculosis-specific antigens (such as ESAT-6, CFP-10) can strongly stimulate IFN-γ release (Haas and Belknap, 2019; Hamada et al., 2021), and that IFN-γ is a key effector molecule against HBV (Maini and Burton, 2019; Wang et al., 2024). We speculate that the higher IFN-γ levels before treatment may have to some extent inhibited HBV replication.Therefore, we propose a testable hypothesis: After the start of anti-tuberculosis treatment, as bacteria are killed and antigen stimulation weakens, the IFN-γ levels rapidly decrease (Adetifa et al., 2010; Vilchèze and Jacobs, 2019), which may lead to the release of inhibition on HBV and trigger viral reactivation. However, it must be pointed out that this study only detected IFN-γ at a single time point and lacks longitudinal data to directly confirm the temporal causal relationship between the decrease in IFN-γand the reactivation of HBV. This mechanism explanation is still inferential and requires future studies to verify through continuous monitoring.

Nevertheless, this potential risk highlights the importance of strict virological monitoring for HBV-TB patients. In our cohort, among untreated individuals, 75.00% showed increased HBV DNA post-treatment, and 81.25% met HBV reactivation criteria—of whom 53.85% progressed to liver injury or failure. We propose the following mechanism: effective anti-tuberculosis treatment reduces mycobacterial antigens, leading to decreased T cell activation and IFN-γ decline. This weakens immune control over HBV, triggering reactivation. Concurrently, drug hepatotoxicity superimposed on the pro-inflammatory microenvironment further elevates DILI risk (Pirmohamed et al., 2002), explaining the poor outcomes in untreated patients (Chen et al., 2018).Similar mechanisms occur in other co-infections. Holmes et al (Holmes et al., 2017). reported HBV reactivation in HBV-HCV patients after HCV clearance, attributed to downregulated interferon signaling (Perrillo, 2017). Our observation of IFN-γ decline with HBV reactivation parallels this, suggesting a broader principle: treating one pathogen may disrupt immune control of HBV by altering the immune microenvironment.This inference is temporally supported by Kim et al (Kim et al., 2016), who found delayed DILI onset (mean 83.3 days) in viral hepatitis patients—consistent with the proposed sequence of IFN-γ decline, HBV reactivation, and subsequent liver injury.

This study has several limitations. First, as a retrospective single-center study, the sample size was limited, particularly for the immunological analysis which was based on a small matched subgroup with limited statistical power and potential selection bias. Importantly, the two comparison groups were not matched according to HBV infection phases, which may introduce grouping bias and affect the reliability of the conclusions. Second, due to the retrospective design, we lacked longitudinal data on IFN-γ dynamics and its correlation with HBV DNA after anti-tuberculosis treatment; therefore, the proposed mechanistic link between declining IFN-γ and HBV reactivation was inferred from cross-sectional data and literature, and requires validation in prospective studies with serial measurements. Third, several exploratory comparisons were conducted without formal adjustment for multiple testing; thus, the findings should be regarded as preliminary and need confirmation in independent cohorts. Fourth, this study only included HBsAg-positive patients; those with isolated HBc antibody positivity (HBsAg-negative, HBcAb-positive)—who are also at risk of HBV reactivation—were not enrolled. Although current guidelines suggest a relatively low risk in this population under standard anti-tuberculosis regimens, their exclusion limits the completeness of our analysis, and future studies should include them to better understand the full spectrum of reactivation risk.

To sum up, while previous studies have mostly attributed the high incidence of severe drug-induced liver injury in HBV-TB co-infected individuals to the direct hepatotoxicity of anti-tuberculosis drugs, or only briefly mentioned possible HBV reactivation (Chinese Medical Association Tuberculosis Branch, 2024; World Health Organization, 2025), this study provides clinical and immunological data supporting an alternative mechanism. We observed that before treatment, patients exhibited high IFN-γlevels, potentially reflecting tuberculosis antigen-driven immune activation coincident with suppressed HBV replication.Following effective anti-tuberculosis therapy, the decrease in IFN-γ levels was associated with HBV reactivation and subsequent liver damage. This temporal pattern is consistent with the hypothesis that relief of immune pressure following tuberculosis antigen clearance may contribute to viral reactivation. Given these observations, current guidelines which manage antiviral treatment based solely on criteria for isolated HBV infection may be insufficient to prevent serious events (You et al., 2023; Chinese Medical Association Tuberculosis Branch, 2024). Therefore, our findings reinforce the recommendation for routine HBsAg screening prior to anti-tuberculosis therapy. Furthermore, considering the risk pattern observed, initiating preventive anti-HBV treatment for all HBsAg-positive individuals, regardless of conventional baseline viral load thresholds, warrants serious clinical consideration.

## Data Availability

The raw data supporting the conclusions of this article will be made available by the authors, without undue reservation.

## Glossary

ALT: Alanine aminotransferase
AST: Aspartate aminotransferase
ALP: Alkaline phosphatase
DILI: Drug-induced liver injury
HBsAg: Hepatitis B virus surface antigen
HBV: Hepatitis B virus
HBVDNA: Hepatitis B Virus Deoxyribonucleic Acid
HBV-R: Hepatitis B Virus Reactivation
TB: Tuberculosis
HCV: hepatitis C virus
WBC: White blood cell
Lym: lymphocyte
Mon: Monocyte
Neu: Neutrophil
RBC: Red Blood Cell
PLT: Platelet
NK: Natural killer
IL: Inerleukin
TNF: Tumor necrosis factor
WHO: World health organization
ULN: Upper limit of normal
AFB: Acid-Fast Bacillus
BALF: Bronchoalveolar Lavage Fluid
ATT: Anti-tuberculosis Treatment
Anti-HBV: Anti-Hepatitis B
DAAs: direct-acting antivirals
